# Probiotic potential of *Lacticaseibacillus rhamnosus* VHProbi M15 on sucralfate-induced constipation in mice

**DOI:** 10.1038/s41598-024-51497-7

**Published:** 2024-01-11

**Authors:** Shumin Cheng, Hongchang Cui, Jingyan Zhang, Qian Wang, Zhi Duan

**Affiliations:** Qingdao Vland Biotech Group Co., Ltd., Qingdao, China

**Keywords:** Applied microbiology, Constipation

## Abstract

The main objective of this study was to investigate the potential probiotic properties of *Lacticaseibacillus rhamnosus* VHProbi®M15 (M15). This study examined the effects of M15 on sucralfate-induced constipation in a mouse model. The BALB/c mice were randomly divided into four groups: the normal group (NOR) was without any treatment, while the constipation (CON), phenolphthalein (PHE), and probiotic (PRO) treatment groups were fed with sucralfate until the appearance of constipation symptoms. Afterward, the NOR and CON groups were given 1 ml saline orally every day until the end of the experiment; the PHE and PRO groups were given phenolphthalein or M15 suspension in 1 ml orally, respectively. Compared with the CON group, the fecal water content and intestinal peristalsis improved in the PRO group. Here, intake of M15 effectively attenuated sucralfate-induced constipation, recuperated colonic epithelial integrity, and increased serum levels of gastrointestinal excitatory neurotransmitters (motilin, gastrin, substance P). Analysis of the intestinal microbiota of mice by 16S rRNA metagenomic revealed an increase in the relative abundance of Bacteroides and a decrease in Sclerotinia, Verrucosa and Proteus in the PRO group. Compared with the CON group, the constipation-induced intestinal microecological changes were partially recovered in the PHE and PRO groups. These results demonstrate that M15 enhanced gastrointestinal transit and alleviated in mice with sucralfate-induced constipation.

## Introduction

Constipation, a common gastrointestinal symptom, affects all ages of humans and may lead to gastrointestinal diseases such as colorectal cancer and irritable bowel syndrome^1^. Standard treatments, such as laxatives, help achieve normal defecation habits, that is, pain-free soft defecation. However, laxatives have several side effects, such as arrhythmia, abdominal cramps, and myocardial infarction^2^. Therefore, it is necessary to treat chronic constipation more gently and effectively.

Probiotics have great prospects as a treatment option for chronic constipation. There are several possible biological mechanisms by which probiotics relieve constipation, including restoring balance after intestinal disorders, altering intestinal sensations and gut microbe metabolites, and regulating the lumen environment^3^. Li et al.^1^ showed that probiotics can increase the amount of short-chain fatty acids (SCFAs), which affects colon peristalsis relieving constipation. Chen et al.^4^ showed that probiotics produced SCFAs reduce intestinal pH and colon transit time. In addition, probiotics-induced changes in intestinal flora can also alleviate constipation symptoms^5^. Presently, lactic acid bacteria (LAB) are one of the most popular probiotics that have been in use for a long time and have no reported risks in humans. Therefore, the development of new LAB probiotic strains is a safe and promising path for the treatment of constipation.

M15, a potential LAB probiotic strain, was identified in the breast milk of those who did not take probiotic products within 6 months. M15 exhibited excellent tolerance against simulated gastric juice and bile salts. This study examined the effects of M15 on constipation and intestinal microflora in mice. We hypothesized that M15 supplementation can enhance intestinal motility and inhibit constipation symptoms. Accordingly, we extracted mice fecal DNA for 16S rRNA high-throughput sequencing and analyzed the structural change in intestinal flora after M15 treatment. Redundancy analysis (RDA) revealed that mice intestinal flora was significantly changed in different treatment groups. Our results highlight the positive effect of M15 on constipation through changes in intestinal motility and flora in mice.

## Results

### Gastrointestinal viability of M15

Survival tests revealed that M15 had a survival percentage of 99.8% after 3 h in artificial gastric juice and 71.4% after 3 h in artificial intestinal juice, respectively. Concisely, the results indicated that M15 have a good survival percentage in simulated gastric and intestinal fluids. The Caco-2 cell line was originally isolated from the human colon adenocarcinoma and is frequently used as an ex vivo model of the intestinal epithelium to study bacterial adhesion properties^6^. M15 adhesion to Caco-2 cells was observed by microscopy. Twenty microscopy images were randomly selected to count the number of M15 and Caco-2 cells. The results showed that the adhesion percentage of M15 on Caco-2 cells was 130%, indicating that M15 has an ability to adhere to Caco-2 cells in vitro and colonize the intestine.

### M15 increased fecal water content in constipated mice

**Figure 1:**
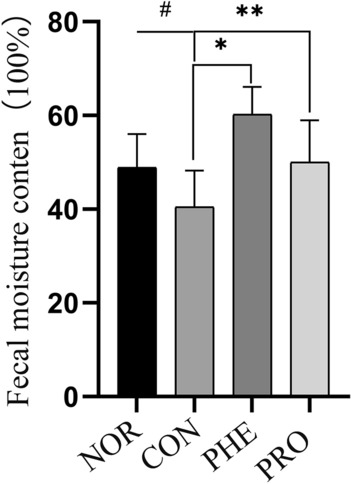
M15 increased fecal water content in constipated mice. Fecal water content in normal (NOR) constipation (CON), phenolphthalein (PHE), and probiotic (PRO:M15) groups; ^#^p < 0.05 compared with NOR; *p < 0.05 compared with CON; and **p < 0.05 compared with PRO.

The water content in feces is related to their hardness and indicates the grade of constipation^7^. The fecal water content in respective mice is shown in Fig. 1. Sucralfate administration significantly reduced the fecal water content in CON mice compared with NOR mice (p < 0.05), indicating the successful establishment of the mice constipation model. Compared with the CON group, the fecal water content in the PRO group was significantly higher, which was almost equal to the NOR group but slightly lower than the PHE group. This data indicated that M15 relieved constipation by increasing fecal water content in mice.

### M15 increased intestinal transit rate (GI) and peristalsis in constipated mice

**Figure 2:**
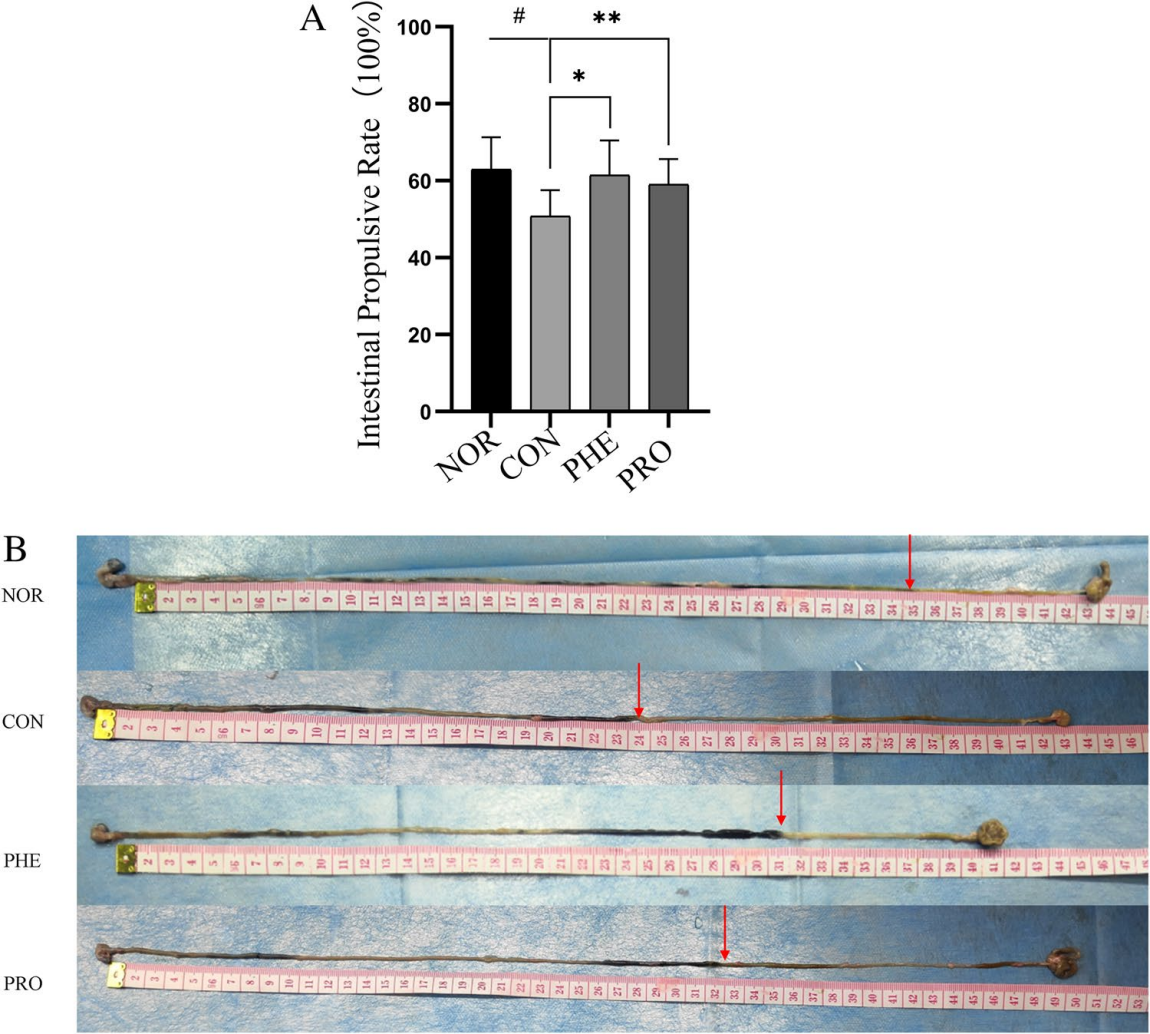
(**A**) M15 improved intestinal propulsive rate in constipated mice. The activated carbon propulsion rate in normal (NOR) constipation (CON), phenolphthalein (PHE), and probiotic (PRO:M15) groups. ^#^p < 0.05 compared with NOR; *p < 0.05 compared with CON; and **p < 0.05 compared with PRO. (**B**) M15 improved intestinal transit length in constipated mice. Active carbon propulsion in normal (NOR) constipation (CON), phenolphthalein (PHE), and probiotic (PRO:M15) groups. The red arrows indicate the peristalsis distance of activated carbon in the small intestines of corresponding mice.

The transportation length of the small intestine is one of the indices to measure the total transportation capacity of the small intestine^8^. The GI transit rate was measured to evaluate the effect of M15 on intestinal peristalsis. Compared with the NOR group, the GI transit rate of the CON group was significantly lower (p < 0.05, Fig. 2A), indicating the establishment of the constipation model. However, M15 supplementation significantly increased the GI transit rate in the PRO group (p < 0.05), almost to the normal level but slightly lower than the PHE group. In all, M15 promoted intestinal peristalsis in constipated mice. Compare with the NOR group, the intestinal transmission length was significantly shorter in other groups (p < 0.05, Fig. 2B) as tested by charcoal movement. However, the intestinal transport length of the PRO group was longer than the CON group. According to the intestinal anatomy, the peristalsis distance was the shortest in the CON group, followed by the PHE group, PRO group, and then the NOR group.

### M15 improved fecal SCFAs levels

As shown in Table 1, The contents of acetic, propionic, butyric, and total organic acids in the feces of all four groups were relatively stable before the establishment of the constipation model by sucralfate (the first 14 days). There was no significant difference in SCFAs concentration between the CON and NOR mice on the 7th and 14th days. However, on the 21st day, the contents of acetic and total acids significantly decreased in CON mice than those in NOR mice (p < 0.001); the acetic acid amounts increased after treatment with M15 in PRO mice (p < 0.05) and became comparable to NOR mice but slightly lower than PHE mice. Likewise, the content of total organic acid also increased (p < 0.05), which was similar to that in NOR and PHE mice.

**Table 1:** Comparison of SCFA content in stool of mice in each group.

Group	Time (days)	Acetic acid (mg/kg)	Propionic acid (mg/kg)	Butyric acid (mg/kg)	Total organic acid (mg/kg)
NOR	7	2470 ± 228.04^ab^	485.33 ± 74.41^acd^	576.17 ± 258.9^ab^	3531.67 ± 385.76^abc^
	14	2550 ± 386.65^c^	456.5 ± 98.94^cd^	554.5 ± 281.94^ab^	3811.67 ± 634.6^a^
	21	2363.33 ± 213.12^c^	478 ± 73.68^a^	545 ± 90.98^bc^	3655 ± 288.6^a^
CON	7	2738.33 ± 468.63^a^	540.5 ± 161.59^acd^	633.33 ± 202.4^ab^	3911.67 ± 630.38^ab^
	14	2596.67 ± 345.14^c^	510.83 ± 92.09^acd^	643.5 ± 278.57^ab^	3951.67 ± 591.65^a^
	21	963 ± 191.64^d^	320.4 ± 155.81^ef^	215.17 ± 127.86^c^	1499.5 ± 414.53^d^
PRO	7	2202 ± 689.91^c^	579.6 ± 146.67^ac^	631.6 ± 346.53^ab^	4040 ± 1153.76^a^
	14	3054 ± 370.17^a^	583.2 ± 164.87^acd^	630.2 ± 292.17^ab^	3960 ± 728.48^ab^
	21	2017.8 ± 240.98^c^	272.2 ± 168.61^ef^	192.06 ± 86.46^d^	3344.6 ± 461.46^bc^
PHE	7	2866.67 ± 394.66^b^	480 ± 57.43^bcd^	571.33 ± 186.36^b^	3888.33 ± 367.35^a^
	14	2682 ± 560.98^b^	498.4 ± 59.51^bcd^	568.6 ± 634.42^b^	4036 ± 1160.82^a^
	21	2779 ± 123.56^b^	258.4 ± 119.29^ef^	175.2 ± 82.48^d^	3462.5 ± 563.63^bc^

Results are expressed as the means ± SD (n = 6).

*NOR* normal group, *CON* constipation group, *PHE* phenolphthalein treatment group, *PRO* probiotic treatment group.

^a^p < 0.05 compared with NOR.

^b^p < 0.05 compared with CON.

^c^p < 0.05 compared with PHE.

^d^p < 0.05 compared with PRO.

### M15 affects serum levels of gastrointestinal regulatory-related peptides

**Figure 3:**
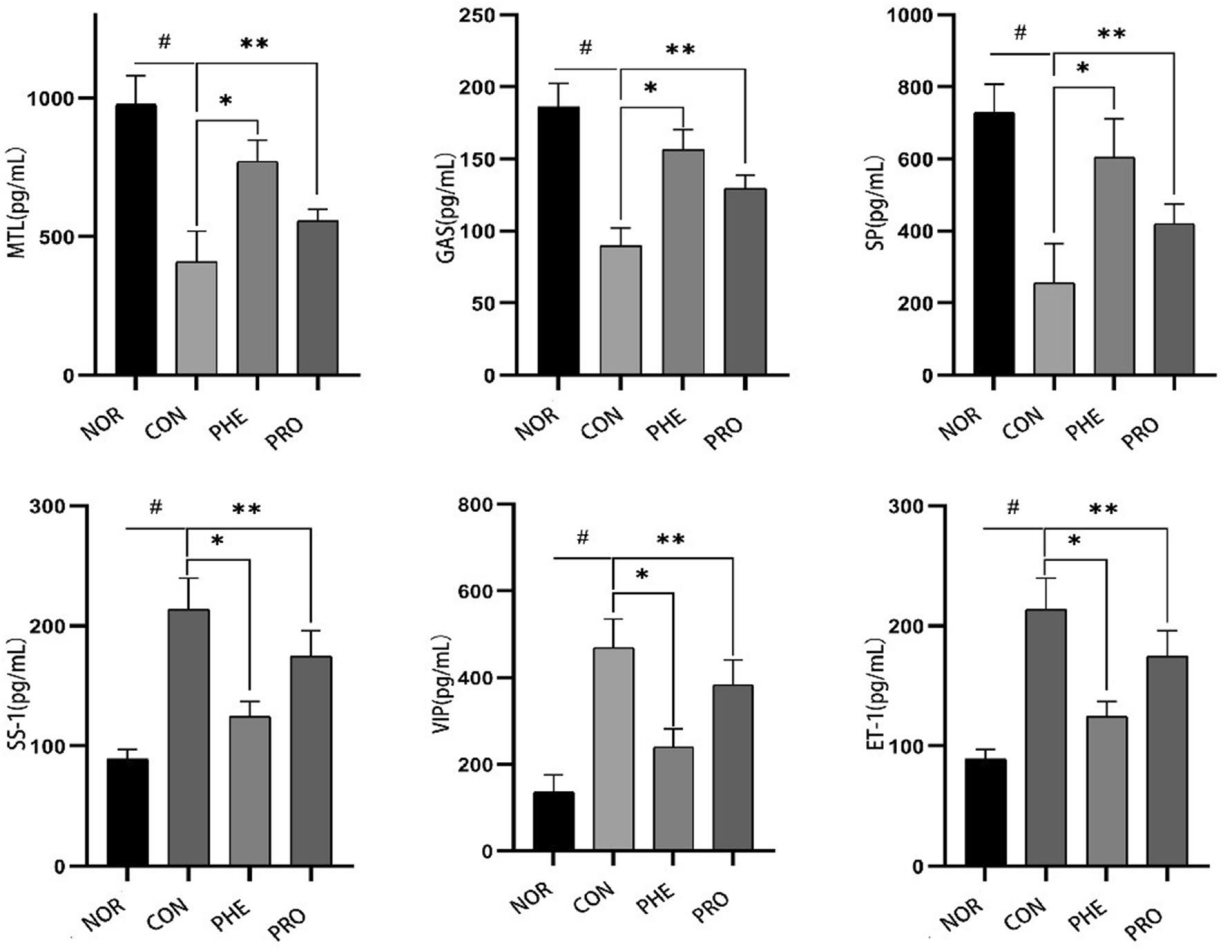
M15 regulated the serum levels of gastrointestinal regulatory peptides. The concentration of gastrointestinal regulatory peptides in normal (NOR) constipation (CON), phenolphthalein (PHE), and probiotic (PRO; M15) groups. ET-1, endothelin; Gas, gastrin; VIP, vasoactive intestinal peptide; SS, somatostatin; MTL, motilin; gastrin; SP, substance P; * the different number of tags in the same row denote significant differences (p < 0.05). Values presented are the mean ± standard deviation (average value of 6 mice in each group).

The effects of M15 on constipation were further evaluated by measurement of serum parameters in the experimental mice, including MTL, Gas, SP, ET-1, SS, and VIP. As shown in Fig. 3**,** compared with the NOR group, the serum levels of MTL, GAS, and SP were significantly decreased (P < 0.01), and that of SS, ET-1, and VIP were significantly increased in the CON group (P < 0.01). However, the trend reversed in the PRO and PHE groups, i.e., MTL, GAS, and SP increased (P < 0.01), while SS, ET-1, and VIP decreased compared with the CON group (P < 0.01). In all, these results thereby indicated that M15 could stimulate the release of gastrointestinal regulatory-related excitatory neurotransmitters.

### M15 supplementation alleviated constipation-induced pathological implications of the small intestine

**Figure 4:**
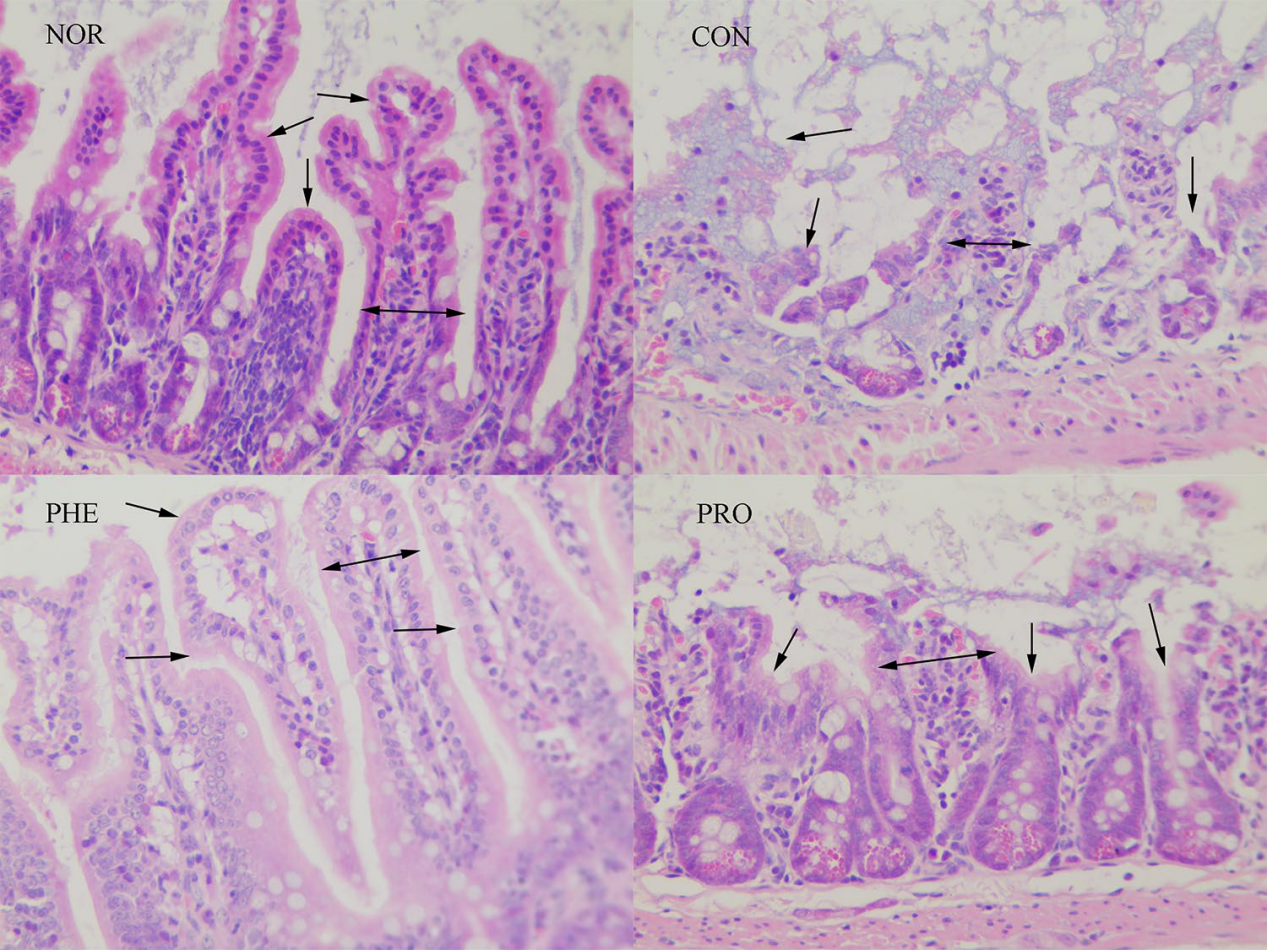
M15 supplementation alleviated constipation-induced pathological implications of the small intestine. Intestinal tissue sections were observed at ×100  using a light microscope. Arrow marks show inflammatory infiltrations. *NOR* normal group, *CON* constipation group, *PHE* phenolphthalein treatment group, *PRO* probiotic treatment group.

The effect of M15 on constipation-induced changes in the distal colon tissue of mice is shown in Fig. 4. The NOR group showed the normal morphological characteristics of epithelial cells after H&E staining of colon tissues; it exhibited well-defined crypts and goblet cells, and the thick layers of colon tissues. In the CON group, intestinal villi were severely broken, the intestinal wall was damaged and the infiltration of inflammatory cells was prevalent. However, the PRO group showed serious signs of recovery; the rupture of intestinal villi and the injury of the intestinal lining were alleviated, intestinal villi became slightly edematous and only a small amount of intestinal villi remained necrotic. This data showed that both M15 and polyphenols effectively inhibited intestinal villi destruction, mucosal ulcer, and colon inflammation.

### M15 restored intestinal flora in constipated mice

**Figure 5:**
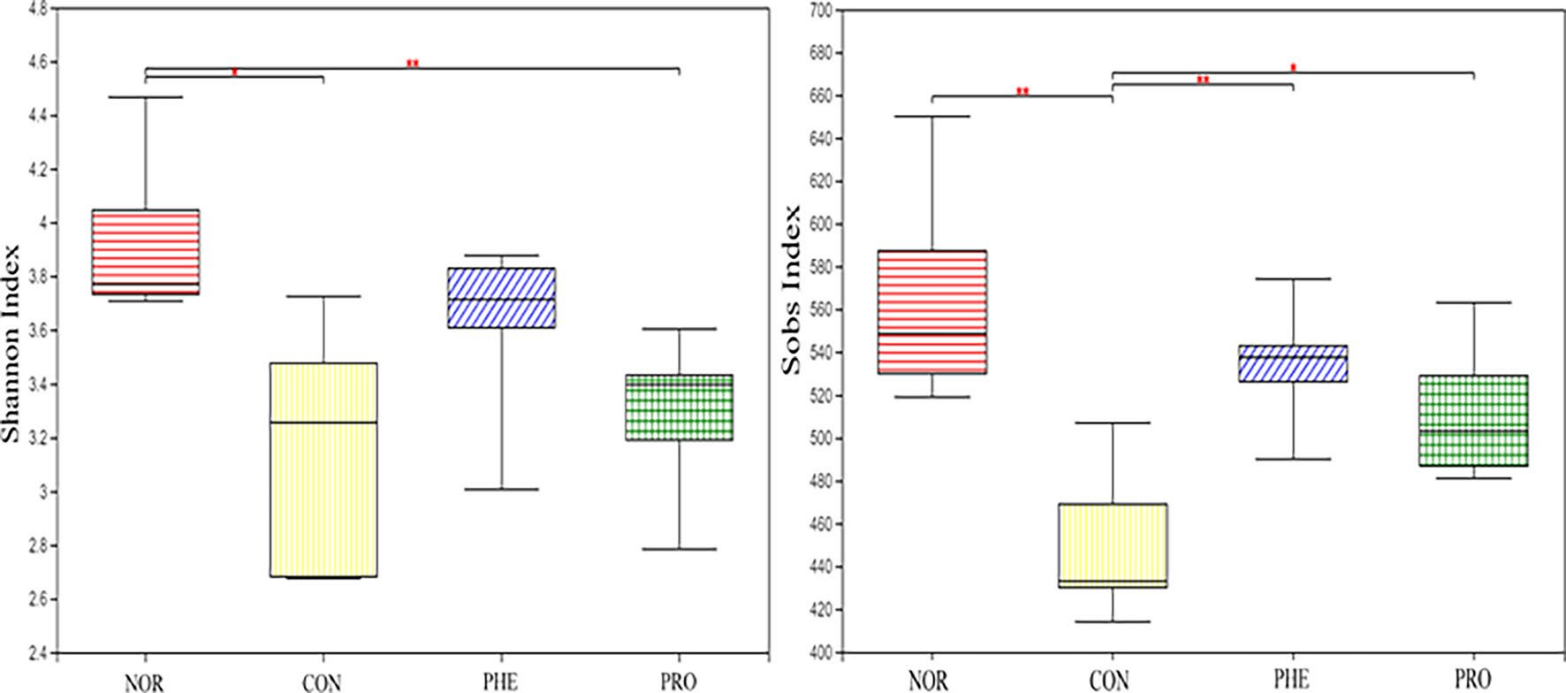
The difference in α diversity index of fecal samples in respective mice. *NOR* normal group, *CON* constipation group, *PHE* phenolphthalein treatment group, *PRO* probiotic treatment group.

The changes in the intestinal flora in mice were mainly detected by the changes in the bacteria in their feces. How do mouse intestinal flora change after constipation, and after supplementation with M15? To explore the effect of M15 on the intestinal microbiome composition of mice, 16S rRNA sequencing was performed by Illumina Hiseq. In this study, 24 mouse feces samples were collected. In total, 2,350,178 effective sequences of 16S rDNA with an average length of 252 bp were generated. The representative sequences of all sequences were clustered based on 97% sequence similarity. The Sobs and Shannon indices were used to reflect the richness and diversity of microbial communities in different samples, respectively. Compared with the NOR group, the Sobs and Shannon indices were significantly decreased in the CON group (Fig. 5**;** P < 0.05), indicating that constipation decreased the richness and diversity of microbial species. However, compared with the CON group, the Sobs index improved in PHE and PRO groups (PRO group, P < 0.01; PHE group, P < 0.05); the Shannon index increased too but the difference was not significant. These results showed that M15 supplementation improved species richness and diversity in CON mice.

**Figure 6:**
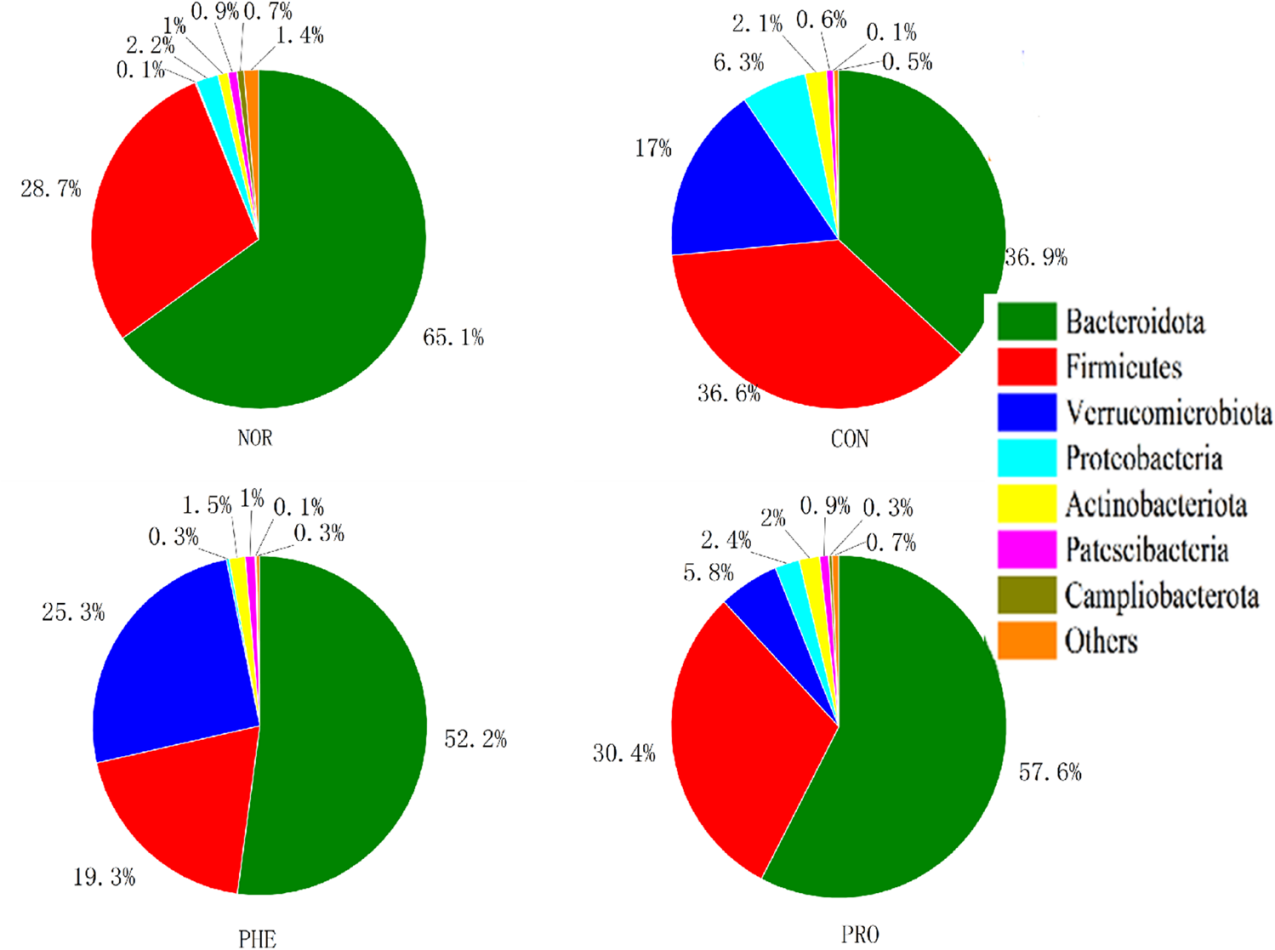
Relative community composition analysis in fecal samples of respective mice at the phylum level. *NOR* normal group, *CON* constipation group, *PHE* phenolphthalein treatment group, *PRO* probiotic treatment group.

When the single operational taxa (OTUs) were analyzed at the phylum level (Fig. 6), we found that the mice intestinal flora was mainly composed of Bacteroides, Firmicutes, Verrucomicrobia, Proteobacteria, and Actinomycetes. Notably. Compared with the NOR group, we found that Firmicutes, Verrucomicrobia, Proteobacteria, and Actinomycetes were increased but Bacteroides were decreased in the feces of CON mice. Firmicutes and Proteobacteria were significantly decreased, while Verrucosa was significantly increased in the PHE group feces. In the PRO group samples, Bacteroides increased, while Firmicutes, Verrucosa, and Proteobacteria decreased; the phylum species composition of the PRO group became similar to NOR mice. The relative abundance of Verrucomicrobia was significantly reduced in the PRO group compared to the PHE group, while the relative levels of Bacteroides were increased. The abundance ratio of firmicutes to Bacteroidetes reappeared in mice after PRO administration. This indicated that M15 supplementation in CON mice restored normal flora composition at the phylum level as in NOR mice.

**Figure 7:**
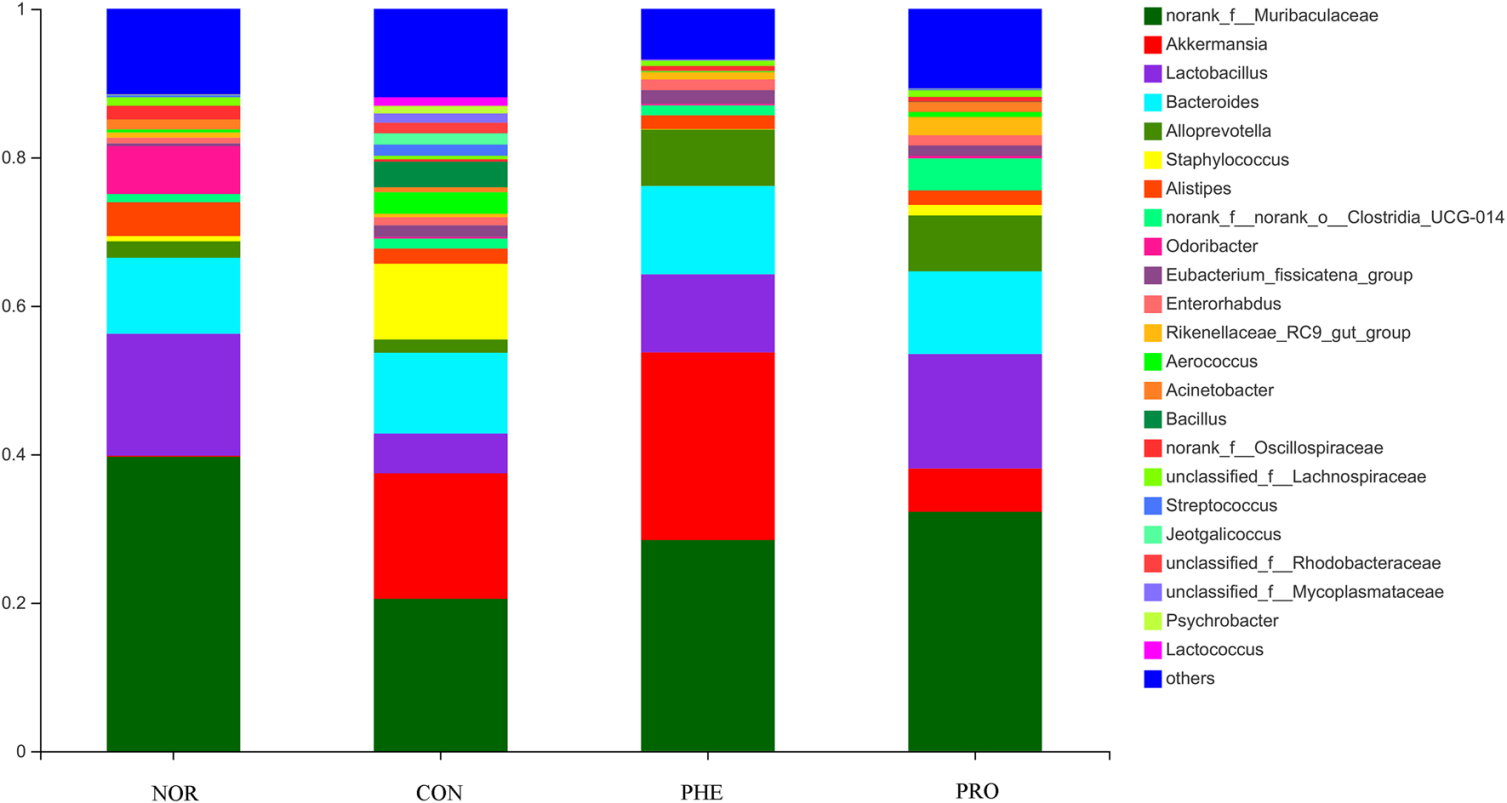
The histogram of community composition analysis in fecal samples at the genus level. *NOR* normal group, *CON* constipation group, *PHE* phenolphthalein treatment group, *PRO* probiotic treatment group.

At the genus level, compared with the NOR group, the abundance of Lactobacillus was significantly decreased while that of Akkemansia and Streptococcus was increased in the CON group (Fig. 7). In the PHE group, Lactobacilli were decreased while Akkemansia and Alloprevotella were increased significantly. Compared with the CON group, Lactobacillus, and Alloprevotella were increased, while Akkemansia and Streptococcus were decreased in the PRO group. Compared with the PHE group, Lactobacillus was significantly increased and Akkemansia was significantly decreased in the PRO group.

**Figure 8:**
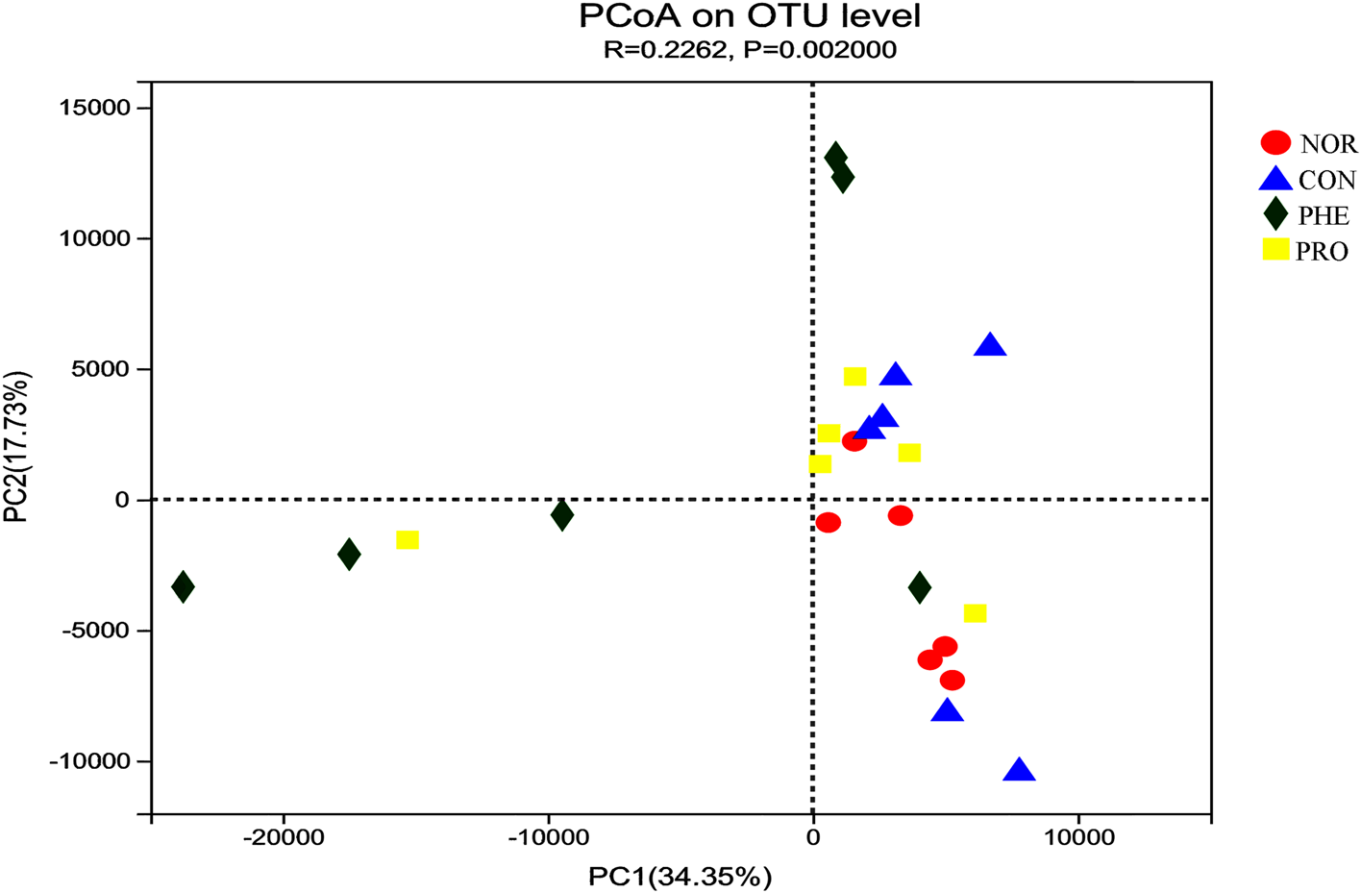
Principal coordinates analysis (PCoA) plot based on unweighted UniFrac metric. Each colored symbol represents the composition of the fecal microbiota in one mouse. *NOR* normal group, *CON* constipation group, *PHE* phenolphthalein treatment group, *PRO* probiotic treatment group.

The β diversity analysis of fecal samples is shown in Fig. 8**.** PCoA coordinate analysis revealed that the mice intestinal flora structure differed between the NOR and CON groups, and PHE and PRO groups, while NOR and PRO groups showed some similarities. CON and NOR groups were significantly separated, indicating that intestinal microbiota composition had changed in the CON group. PRO and NOR groups were closely clustered, suggesting that PRO treatment could restore the composition of intestinal microbiota to a certain extent. Overall, the above results indicated that M15 somewhat restored normal intestinal flora in constipated mice.

## Discussion

Probiotics are living microorganisms that can positively impact human health when ingested in sufficient quantities. Gut microbiota plays an important role in gastrointestinal homeostasis and functions. With the advancement of science and technology, food has become more refined, but sleep and exercise have been greatly reduced. These social changes have increased constipation symptoms such as prolonged defecation and hard stool in people. At present, Lactobacillus, Bifidobacterium, Bacillus, and Streptococcus have been used as probiotics in humans, which play a positive role in many aspects of the human body. However, most probiotics lose activity during digestion. Survival in gastric and intestinal juices is the basic requirement for probiotics to colonize the gastrointestinal tract, and then play a role in the target site. We screened the M15 strain from fresh breast milk, which showed good survival in artificial gastric (99.8%) juice, which means M15 can survive in a harsh gastrointestinal environment. In addition, the Caco-2 cell line isolated from human colon adenocarcinoma was used as an in vitro model of the intestinal epithelium to study M15 adhesion to the intestine. The number of M15 adhered to Caco-2 cells was counted by microscopy counting; we found a large number of M15 adhered to the cell surface. M15 showed good adhesion properties indicating that it is well capable of colonizing the colon, a prerequisite of a good probiotic. The high survival percentage in the gastrointestinal setting is M15's greatest advantage as a probiotic to relieve constipation symptoms.M15 is a strain of *Lactobacillus rhamnosus*, which is different from *B. animalis* VHProbi YB11^9^ published by CUI. M15 has better growth ability and gastrointestinal fluid tolerance than YB11, and has a more significant effect on constipation. In addition, M15 has more potential for application, as it provides in-depth study of the effects of probiotics on the improvement of intestinal flora.

Sucralfate is mainly used for gastric mucosal injury in clinical practice. Also, it can induce constipation in mice by inhibiting intestinal motility and increasing intestinal water absorption^10^. In this study, the mice constipation model was established by oral administration of sucralfate. Fecal water content and intestinal transit ratio are commonly used to evaluate constipation. The slower the intestine peristalsis, the longer feces remain in the intestine. This leads to more water absorption in the intestine, leaving less water content in feces. Meanwhile, excessively dry feces may also cause damage to the intestinal lining villi^11^. On the contrary, good intestinal peristalsis improves the fecal water content, relieving constipation symptoms. Compared with the NOR group, the CON group showed significantly decreased water content, decreased intestinal passage rate, and severe intestinal lining damage, while the PRO and PHE groups showed improved fecal water content and intestinal passage rate and reduced intestinal villi damage compared with the CON group. These results suggested that M15 can relieve constipation by promoting fecal moisture content and peristalsis.

SCFAs are metabolites of intestinal bacteria and are closely related to the peristaltic efficiency of the intestine^12^. SCFAs can stimulate intestinal peristalsis and increase osmotic pressure, thus increasing the water content in the intestine^13^. Low intestinal pH promotes the competitive binding of SCFAs to receptors in intestinal epithelial cells or mucosa, improving intestinal flora in patients with constipation^14^. A previous study^15^demonstrated that probiotics can improve defecation by increasing the colon content of SCFAs and in turn softening fecal stools in constipation model animals. Li & Nie ^1^also showed that SCFAs are essential for the integrity of the colonic epithelium: they stimulate the proliferation of epithelial colonic cells and have a protective effect on the epidermis of the large intestine.Therefore, it can be concluded that the improvement of constipation is positively correlated with the increase in SCFAs. M15 relieved constipation symptoms by increasing the levels of SCFAs in PRO mice.

Serum levels of gastrointestinal regulatory-related neurotransmitters are closely related to intestinal peristalsis. MTL, GAS, and SP are excitatory regulatory peptides, and increased levels of these peptides promote promote intestinal water and electrolyte transport, regulate gastrointestinal movement, accelerate GI time, and relieve constipation, whereas, SS, ET-1, and VIP are inhibitory regulatory peptides, and decreased levels of these peptides in the serum relax the sphincter and play an important role in cardiovascular, neuroendocrine and other aspects^14^. MTL influences the transport of water and electrolytes, promotes gastric contractions and small intestine segmental movement, accelerates intestinal transfer time, and increases colon movement. Gas stimulates the secretion of gastric acid and pepsinogen and promotes the growth of digestive tract mucosa, the contraction of the GI smooth muscle, and the relaxation of the pyloric sphincter. SP adjusts the contraction of the GI tract, intestinal motility, and gastric acid secretion. VIP is a polypeptide composed of 28 amino acids whose function is to relax the GI tract and GI sphincter, and it significantly promotes the colon cancer induced by carcinogens in mice. VIP is an important factor in the production of descending inhibition, resulting in slow transmission^16^. The combined action of excitatory and inhibitory regulatory peptides accelerates intestinal emptying. In the present study, the CON group showed obvious constipation-related symptoms, including a significant decrease in MTL, Gas, and SP levels, and an increase in ET-1, SS, and VIP levels, but M15 increased serum levels of MTL, GAS and SP in CON mice (P < 0.05, Fig. 3). This suggested that M15 may reduce the severity of constipation by modulating neuropeptide levels.

Recently, the change in intestinal microbiome composition has been closely associated with the pathophysiology of constipation^7,17^. Notably, there are many reports that probiotics not only benefit the health of the intestinal tract but also regulate the intestinal flora of the host^18^. Therefore, in this study, we studied the effect of M15 on the intestinal flora of CON mice to explore the specific mechanisms through which M15 relieved constipation symptoms in CON mice. Alpha diversity is used to analyze the richness of gut microbiota. Here, we evaluated microbial community diversity using the Shannon index at the OTU level. The regulatory effect of M15 on microbial diversity in CON mice is shown in Fig. 5. Notably, M15 partially restored intestinal bacterial diversity in CON mice. It has been reported that alpha diversity of fecal flora is related to fecal water concentration. A study showed that with the decrease in fecal moisture, community diversity decreased and reached the lowest value among individuals with diarrhea^19^. The water content of PHE was greater than that of PRO in this study (Figs. 1, 2), while the results from Shannon's index showed that the community diversity of the PHE group was higher than that of the PRO and CON groups (Fig. 5), which was in agreement with the results of previous reports..

Fu et al.^20^ performed 16S rDNA sequencing of the fecal samples from constipated patients and found that the abundance and diversity of intestinal flora were significantly lower in constipated patients than in healthy people. The abundances of Firmicutes and Bacteroidetes have been used as determinants of human constipation^21^. At the phylum level, for humans and mice, the overall composition of microbiota in feces is mainly dominated by Firmicutes and Bacteroidetes. Many studies have shown that constipation is associated with changes in the gut microbiota. On the one hand, the activity of gut microbes can promote intestinal motility by influencing the concentration of SCFAs. It has been reported that LAB in the intestine can change intestinal motility through SCFAs, accelerating stool formation, promoting defecation, and reducing constipation symptoms^22^. SCFAs are absorbed and can reduce the osmotic pressure of intestinal cells. Such intestinal cells hold enough water and do not absorb more water from the stool to be excreted (Fig. 3). Bacteroidetes can produce propionic acid, and butyric acid levels are positively correlated with the relative abundance of Firmicutes.Both propionic acid and butyric acid levels play an important role in relieving constipation.On the other hand, the abundance of Bacteroides and Firmicutes can improve intestinal motility by provoking the release of 5-HT or by promoting cholinergic pathways. Similarly, in our study (Fig. 7), compared with the NOR group, the proportion of Firmicutes increased in the CON group, while the number of Sclerotinia, Verrucosa and Proteus decreased. Sclerotinia, Verrucosa and Proteus have been associated with colitis and gastroenteritis, and these species of anaerobes are pathogenic to humans. Overall,the contents of intestinal metabolites are closely related to the composition of intestinal flora.

Wang et al.^11^ found that the number of bifidobacteria and lactobacillus in the feces of constipated patients was significantly lower than that in healthy people. According to species composition analysis at the genus level, we found significant differences between the PHE and PRO groups; Lactobacilli were increased, while Akkemansia were decreased in the PRO group, indicating that phenolphthalein and probiotics had different regulatory effects on the intestinal flora of model CON mice. Additionally, *Lactobacillus* has been reported to suppress inflammation and promote gastrointestinal motility, improving constipation^23^. The associated underlying mechanisms of Lactobacillus species ameliorating constipation symptoms are likely through the increase in fecal softness, stool shaping, and defecation frequency^24^. The increase in lactobacillus in the PRO group suggested that M15 had a positive effect against constipation in mice. The β diversity analysis is mainly used to study species diversity among different samples and can be represented by a PCoA diagram (Fig. 8). We found differences between the intestinal flora compositions of the NOR and CON groups, and the PHE and PRO groups, but the intestinal flora compositions of the NOR and PRO groups were closely similar. This indicated that administration of M15 improved the intestinal flora of CON mice to a certain extent alleviating the damaging effect of sucralfate.

## Conclusion

This study will provide the following valuable data for clinical practice: (1) in the treatment of constipation, M15 has a similar effect as phenolphthalein in improving intestinal peristalsis and increasing fecal moisture content; (2) M15 and phenolphthalein can regulate intestinal flora structure and increase fecal SCFA content; however, compared with phenolphthalein, M15 have different effects on the structure of gut microbiota and the composition of SCFAs in feces. In conclusion, we demonstrated that M15 improved intestinal emptying and modulated gut bacterial populations, thereby alleviating constipation in CON mice. M15 supplementation in CON mice improved fecal water content, intestinal peristalsis, and modulated gut flora and regulatory peptides. The potential therapeutic mechanism of M15 is related to the recovery of intestinal microbial composition, as well as the beneficial regulatory effects of intestinal metabolites such as SCFA. But the specific regulatory substances and regulatory pathways need more research. In all, M15 can be a potential probiotic strategy against constipation.

## Materials and methods

### Chemicals and reagents

2 mg/5 ml sucralfate (Haipu Pharmaceutical, China) was diluted twice in 0.8% normal saline. The solution of activated carbon powder was prepared as follows: 100 g of Arabic gum was boiled in 800 ml of water until the solution turned transparent. Then, the solution was added with 50 g of activated carbon and boiled for another 30 min. Next, the mixture was cooled, diluted to 1000 ml with water, and then stored at 4 °C. The solution was thoroughly mixed before use. The kit for measuring serum neuropeptide levels was purchased from Lewin Bioengineering Institute, Shanghai, China. De Man, Rogosa, and Sharpe agar (MRS) culture medium were from Beijing Luqiao Technology Co., Ltd, China. All the used reagents were of analytical grade.

### Strain and culture condition

M15 was isolated from breast milk, which from locally recruited individuals and informed consent was obtained. M15 was preserved in China Center for Type Culture Collection (Wuhan, China) with the preservation number CCTCC M 2021904. M15 stored in glycerol MRS broth (30% (v/v), − 80 °C) was first cultured in MRS media under the anaerobic condition at 37 °C for 24–48 h and then transferred to fresh MRS media at 2% (v/v) inoculum. M15 needs to be activated at least three times continuously. For the animal experiments, the M15 bacterial suspension was prepared in sterile normal saline at 1 × 10^9^ CFU/ml.

### M15 tolerance test against artificial gastric and intestinal juices

Probiotics should not only survive against the gastric and intestinal juices but also be able to colonize the gut. Accordingly, we tested the survival of M15 against the artificial gastric and intestinal juices as follows. Briefly, the M15 solution was centrifuged for 10 min at 5000 rpm and 4 °C. The obtained supernatant was discarded and the bacterial pellet was suspended in normal saline (10^9^ colony-forming units, CFU/ml). 1 ml of M15 suspension was added to 50 ml of artificial gastric juice (pH 3.0) and after incubation for 3 h at 37 °C, the viable bacteria were counted to find the M15 tolerance. For the tolerance test against the intestinal juice, after the 3 h incubation with the artificial gastric juice from the first step, 1 mL of artificial gastric juice (having M15) was mixed with 24 ml of artificial intestinal juice and the mixture was incubated at 37 °C for 3 h. Afterward, the number of viable bacteria was counted using the GB 4789.35-2016 code.

### Determination of M15 adhesion properties

The adhesion of M15 to Caco-2 (Purchased from North Carolina Bio Corporation) cells was detected according to the method of Coconnier et al.^25^. Briefly, a slide was placed in a six-well plate, then added with 1 ml of cell fluid (2 × 10^5^cells/well) for culture for 24 h. Then, the slide was washed with sterile normal saline to remove unattached Caco-2 cells. 1 ml of the strain suspension (5 × 10^7^ CFU/ml) was added into the well and incubated in a CO_2_ room at 37 °C for 2 h. The unattached bacteria were removed by washing with sterile normal saline. Finally, the slide was fixed in methanol (Aladdin) and examined under a microscope after Gram staining.

### Animal trial

#### Animals and experiment design

Twenty-four female specific-pathogens-free BALB/c mice (age 6–8 weeks, weight 18–22 g) were randomly divided into four groups and kept under adaptive feeding for one week before the experiment (room temperature, 25 ± 2 °C; relative humidity, 50 ± 5%; 12 h light/12 h dark cycle). All mice were fed the usual diet from day 1 to the end of the study (Day 21). The normal group (NOR) was given 1 ml saline solution by intragastric administration until the end of the study (Day 21). To establish the mice constipation model, mice were gavaged 1 ml of sucralfate (50%) in saline solution (Day 7). The constipation model was validated based on the constipation symptoms such as the volume reduction and dryness/hardness of the fecal stools^8,26^. Eighteen constipated mice were divided into three treatment groups (6 each): the constipation group was treated with 0.8% sterilized saline (CON; 1 ml), the probiotic group was treated with M15 suspension (PRO; 1 ml × 10^9^ CFU/ml), and the phenolphthalein group was treated with 1 ml phenolphthalein (PHE; 70 mg/kg body weight). Howarth and Sullivan^27^ found that polyphenols can dredge constipation. Therefore, the polyphenol treatment group was used as a control experiment. All groups were treated with the corresponding dosage once a day via oral gavage for 2 weeks until the end of the study (Day 21). Mice in each group were maintained in single cages. The feces of mice on the 7th, 14th, and 21st days were collected and frozen to detect the content of organic acids in the feces.At the conclusion of the study period, all mice were anesthetised with 4% isoflurane and sacrificed by cervical dislocation.

### Ethics declaration

Breast milk samples in this study were kind gifts from volunteers in China Qingdao and collected according to the guidelines laid down in the Declaration of Helsinki, and informed consent was obtained from all participants.

The BALB/c mice were purchased from the Experimental Animals Center of Qinglongshan (Jiangsu, China).The animal permit number was SCXK (Su) 2017-0001. All procedures were carried out according to protocols approved by the Animal Care and Use Committee of the laboratory animal ethics committee Nanjing BIOGENE Biotech. Co. Ltd. Ethical review number is 202300613105357. All experiments were performed in accordance with relevant guidelines and regulations and reported in accordance with ARRIVE guidelines.

### Fecal water content

On Day 21 (end of the study), fresh feces from all groups were collected into tubes from 10:00 p.m. to 10:00 a.m. Each stool sample was divided into three equal parts and used for the estimation of stool water content and SCFA value. The weight difference between feces before and after drying was determined by the freeze-drying method. The stool moisture content was determined using the formula from Jeon and Choi (2010)^28^

1 $$\mathrm{Fecal\, moisture\, content } \, ({\%})=\frac{\mathrm{wet\, weight}-\mathrm{dry weight}}{\mathrm{wet\, weight}} \times 100$$

#### Intestinal transit ratio

The intestinal transport rate was determined by the following method. Briefly, the animals were fasted for 24 h on Day 21; an aqueous suspension of 3% charcoal in 0.3 ml was orally administered to each animal. After 1 h, mice were euthanized and the whole intestines were collected to measure the transit distance of activated charcoal. The distance covered by the charcoal powder and the total length of the small intestine were measured to calculate the intestinal transport rate as follows:

2 $$\mathrm{Intestinal\, transit\, rate} \, ({\%})=\frac{\mathrm{Distance\, traveled\, by\, the\, charcoal}}{\mathrm{Total\, length\, of\, the\, small\, intestine}} \times 100$$

### Quantitative assay of SCFAs in feces

The concentrations of SCFAs were determined according to previous reports using gas chromatography-mass spectrometry (QP2010 Ultra System, Shimadzu Corporation, Kyoto, Japan) with an Rtx-Wax column (30 × 0.25 × 0.25 m)^29^. Briefly, 200 mg fecal samples were vortexed with 1 mL aqua distillate for 10 min and centrifuged at 5000 rpm at 4 °C for 10 min. Subsequently, 500 µL of the supernatant was filtrated using a 0.22 µm Millipore filter and thoroughly mixed with 50 µL of 50% sulfuric acid solution (v/v). Then 800 µL diethyl ether was used for SCFAs extraction for 20 min and centrifuged at 10,000 rpm at 4 °C. On the 7th, 14th, and 21st days, the contents of acetic, propionic, butyric, and total organic acids were detected in animal feces. The fecal concentration of SCFAs was calculated according to the standard curve.

#### Determination of serum neuropeptides levels

The serum levels of neuropeptides including motilin (MTL), gastrin (Gas), endothelin -1 (ET-1), somatostatin (SS), substance P (SP), and vasoactive intestinal peptide (VIP) were measured using the corresponding enzyme-linked immunosorbent assay (ELISA) kits following the manufacturer's instructions (eBiosource^®^, United States)^30^**.** Blood samples collected from mouse eye sockets were centrifuged to obtain the serum. Then, serum, biotin-labeled secondary antibodies, ELISA reagent, and standard sample were added to the kit and kept for 60 min at 37 °C. The wells were washed twice with PBS solution. Then, color A and B reagents were added and the reaction was incubated at 37 °C for 10 min. Finally, a terminating reagent was added and the sample absorbance was recorded at 450 nm. Then the corresponding neuropeptide concentrations were calculated.

#### Histological analysis

The ileocecal valve was collected from the pyloric entrance of the mouse stomach. After washing the small intestine lumen with saline, the small intestine was fixed in 10% formalin^31^. The tissue was dehydrated in 95% ethanol for 24 h and then treated with xylene. Next, the small intestine was sectioned (5 μm thickness), stained with H&E (hematoxylin–eosin), and observed under a phase contrast microscope at 200× magnification^28^ (BX43, Olympus, Tokyo, Japan).

#### Fecal microbiome profiles by 16S rRNA gene sequencing

After the last gavage on Day 21, stool samples were collected from 10:00 p.m. to 10:00 a.m. Stool DNA was extracted using a Tiangen genomic DNA extraction kit (China) according to the manufacturer’s instructions. The operation method of 16S rRNA gene sequencing was measured according to the method described by Zhao^32^. The high-quality sequences were obtained through the assembly. After sequencing, the sequences with greater than 97% similarity were defined as operational taxons (OTUs) by QIIME software. To obtain the corresponding species classification information, all OTUs were aligned to microbial reference databases from phylum to species. Subsequently, QIIME software was used to generate species richness at every taxonomic level. Principal coordinate analysis (PCoA) was used to analyze the beta diversity of the fecal microbial communities. Chao and Shannon indices were used to evaluate the Alpha diversity index and species richness of samples.

### Statistical analysis

One-way analysis of variance (ANOVA) was used to analyze the statistical difference between the sample means data. Statistical analysis was carried out with SPSS 20.0 software. Data were plotted with GraphPad Prism 8 (Supplementary Information).

### Supplementary Information

Supplementary Information.

## Data Availability

The sequences were deposited in the NCBI Sequence Read Archive under BioProject accession Number: PRJNA1035229 (https://www.ncbi.nlm.nih.gov/sra/PRJNA1035229).The datasets used and/or analysed during this study are available from the corresponding author on reasonable request.The raw data supporting the conclusions of this article will be made available by the authors, without undue reservation.
